# Predicting Postoperative Emergence Delirium From the Heart Rate Variability of Patients Undergoing Elective Cardiac Surgery

**DOI:** 10.7759/cureus.34613

**Published:** 2023-02-03

**Authors:** Maiko Satomoto

**Affiliations:** 1 Anesthesiology, Toho Univesity, Tokyo, JPN

**Keywords:** anesthesia, heart rate variability, mmse, surgery, postoperative delirium

## Abstract

Background and objective

The complication of postoperative delirium is directly linked to prognosis, leading to prolonged hospital stays and an increase in mortality. Since there is no magic medicine that cures delirium, the prevention of its onset is important, and the development of simple tools that enable the early assessment of the risk is valuable. In the previous study, we hypothesized that postoperative delirium could be predicted from heart rate variability (HRV) measured by using an electrocardiogram (ECG) on the day before elective esophageal cancer surgery. HRV is calculated based on the fluctuation of RR intervals on ECG. The preoperative high-frequency (HF) power in delirium patients was significantly lower than that in non-delirium patients. The HF component is considered a reflection of parasympathetic function. In the current study, we evaluated the hypothesis that parasympathetic nerve activity is low in the resting HRV on the night before surgery in patients who go on to develop postoperative delirium. To that end, we recorded resting HRV in patients scheduled for cardiac surgery on the night before surgery. We then compared the HRV between patients with and without delirium in the postoperative intensive care unit (ICU). The Confusion Assessment Method for the ICU (CAM-ICU) was used to diagnose delirium.

Methods

This was a prospective observational study involving patients undergoing elective cardiac surgery. After obtaining approval from the institutional review board, patients aged 65 years and older were enrolled in the study. The day before surgery, a Mini-Mental State Examination (MMSE) was performed. The ECG was used in patients for five minutes. All patients were transferred to the ICU after surgery, and CAM-ICU was measured every eight hours until discharge from the ICU, and positive patients were diagnosed with delirium.

Results

In this study, 14 patients who developed delirium and 22 patients who did not were included in the analysis. The average MMSE score was 27.4, with no patients diagnosed with preoperative dementia. In the analysis of HRV, the HF component was significantly lower in the group with delirium compared to the group without delirium (Mann-Whitney U test, p<0.05).

Conclusion

Based on our findings, in patients with postoperative delirium, the activity of parasympathetic nerves was lower than before surgery, and we concluded that it is possible to predict the onset of postoperative delirium based on preoperative ECG measurement.

## Introduction

Postoperative delirium is a severe complication that prolongs hospital stay and worsens prognosis [1,2]. There is evidence to suggest that postoperative delirium causes postoperative cognitive decline [3] and leads to anxiety among patients. Of note, 80% of elderly patients have been reported to develop postoperative delirium in the intensive care unit (ICU) [4]. In planned surgeries, the patient can be prepared against conditions that can induce delirium, such as electrolyte imbalance and dehydration, before the surgery [5]. However, even if the environment and physical condition are adjusted for scheduled surgery, elderly patients are still prone to delirium, especially those aged 65 years or older, who are considered a high-risk group for delirium. Patients undergoing cardiac surgeries using a heart-lung machine are considered a high-risk group for postoperative delirium [6,7], and they may also suffer from postoperative cognitive dysfunction (POCD) at a high rate.

In our previous study, we investigated the occurrence of postoperative delirium after esophageal cancer surgery and whether it can be predicted by heart rate variability (HRV). And we reported that a decrease in the high-frequency (HF) component of HRV on the day before surgery may predict delirium [8]. Based on this finding, we proposed that if postoperative delirium can be predicted by the non-invasive short-term analysis of HRV, it could be used for the more efficient allocation of limited medical resources.

In this study, we focused on open heart surgery involving cardiopulmonary bypass, such as valve replacement surgery, coronary artery bypass surgery, and great vessel replacement, in which delirium commonly occurs, and examined whether postoperative delirium can be predicted based on HRV in patients aged 65 years or older. For diagnosing postoperative delirium, we used the Confusion Assessment Method for the ICU (CAM-ICU), which is more straightforward and widely used compared to the diagnosis by psychiatrists [9,10].

This article was previously presented as a meeting abstract at the 2022 Euroanaesthesia Annual Scientific Meeting on June 4, 2022 [11].

## Materials and methods

After obtaining IRB approval (M19242, trial registration No. UMIN000036436), we conducted a single-center prospective observational study. Informed consent was obtained in writing. Patients aged between 65 and 88 years with planned open-heart surgery were included in the study. Patients with dementia and continuous atrial fibrillation were excluded. According to the literature, postoperative delirium after open heart surgery occurs in 56% [7] of patients aged 65 years or older; therefore, the required sample size was 14 cases in each group, with a confidence interval of 95% and a margin of error of 5%.

A five-minute resting electrocardiogram (ECG) test was taken in the patient's room in the afternoon of the day before surgery. A three-lead wired ECG was used, which was captured on a computer, and the person operating the ECG confirmed on the spot that the ECG was adequate. MemCalc/Bonaly Light (Suwa Trust GMS, Tokyo, Japan) was used to analyze the frequencies in the ECG. Three patients with atrial fibrillation and flutter complications were excluded. A Mini-Mental State Examination (MMSE) test was also performed at the bedside.

The HRV analysis

The RR interval of the electrocardiogram was plotted for five minutes and analyzed by frequency. The following frequency domain features can be obtained from the power spectrum density of the sampled RR: low-frequency power (LF) and high-frequency power (HF); and the ratio thereof (LF/HF) was calculated. LF is related to sympathetic and parasympathetic nerve activity, while HF correlates with parasympathetic nerve activity. LF/HF ratio reflects sympathetic nerve activity [12].

All patients underwent general anesthesia and surgery and were transferred to the ICU under sedation. The depth of anesthesia was properly controlled with bispectral index (BIS) to avoid deep anesthesia. At our hospital, after transfer to the ICU, if there are no problems with bleeding, breathing, or circulation, we turn off sedation and wake up the patients; hence, extubation takes about four hours to the next morning after surgery. CAM-ICU was measured every eight hours (three times a day) until the patient was discharged from the ICU. Positive CAM-ICU was considered to be delirium.

**Figure 1:**
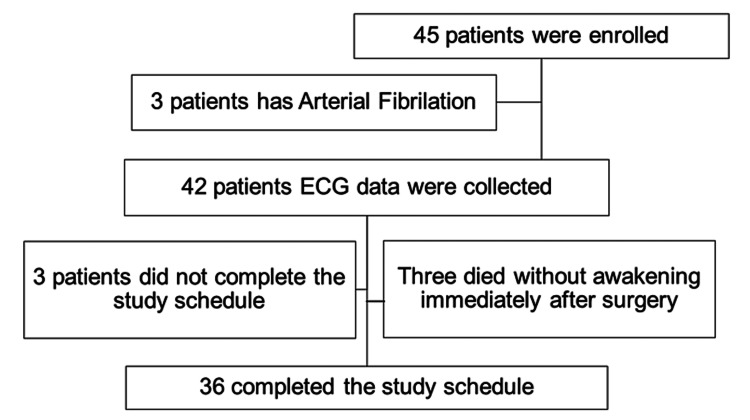
Patient flow diagram

This observational study placed no restrictions on prophylactic or therapeutic medications and on intervention with cardiovascular surgical management during the perioperative period. One patient with a short stenting surgery who did not undergo open-heart surgery and two dialysis patients were excluded from this study because they had to be extubated after removing water so as not to affect the circulation, resulting in a longer postoperative intubation period. Three patients had paroxysmal atrial fibrillation rather than chronic atrial fibrillation and were excluded after the initial inclusion. Three patients passed away the next day or the day after without waking up even once from the anesthesia. Ultimately, the analysis was conducted among 36 patients (Figure 1).

Postoperative analysis was conducted for patients who had come out of sedation and were fully conscious, even if they were intubated. Once the presence of delirium was determined based on CAM-ICU, the patient was considered as having the onset of delirium, even if the delirium improved with treatment or recovery from the medical condition.

Statistical analysis was performed using IBM SPSS Statistics Version 20.0 Media Pack (IBM Corp., Armonk, NY), and data were expressed as mean ± standard deviation (SD). The two groups were compared using the Mann-Whitney U test.

## Results

A total of 42 patients were initially enrolled, and the results of 36 patients were analyzed. Fourteen patients developed delirium (positivity rate: 33%), and 22 did not develop delirium. The patient characteristics are shown in Table 1. There was no significant difference between the delirium group and the non-delirium group in terms of age, sex, duration of surgery, duration of anesthesia, preoperative MMSE value, American Society of Anesthesiologists (ASA) physical status, and Charlson Comorbidity Index. However, the length of stay in the ICU and cardiopulmonary bypass time were longer in the delirium group compared to the non-delirium group (T-test, p<0.05).

**Table 1:** Preoperative and intraoperative characteristics of patients in the delirium and non-delirium groups *P<0.05 MMSE: Mini-Mental State Examination; ASA: American Society of Anesthesiologists; SD: standard deviation

Characteristic	Delirium group (n=14)	Non-delirium group (n=22)
Age, years, mean (SD)	75.0 (7)	73.6 (5)
Men, n (%)	10 (71)	14 (64)
Duration of surgery, hours, mean (SD)	8.6 (2.7)	6.9 (1.8)
Duration of anesthesia, hours, mean (SD)	10.2 (2.8)	8.3 (2.0)
Intraoperative bleeding, g, mean (SD)	890 (841)	966 (805)
Preoperative MMSE score, mean (SD)	26 (4.0)	27.5 (2.0)
ASA physical status, mean (SD)	4 (0)	4 (0)
Charlson Comorbidity Index, mean (SD)	3.8 (1.8)	1.7 (1.7)
Length of stay in ICU, days, mean (SD)	7.9 (5.1)*	3.6 (1.2)*
Cardiopulmonary bypass time, hours, mean (SD)	4.7 (1.5)*	3.4 (1.0)*

The average MMSE score was 27.4, with no patients diagnosed with preoperative dementia. In the analysis of HRV, the HF component was significantly lower in the group with delirium than in that without delirium (Mann-Whitney U test, p<0.05) (Table 2).

**Table 2:** Comparison of preoperative HRV data between delirium and non-delirium groups HRV: heart rate variability; LF: low-frequency; HF: high-frequency; LH/HF: ratio of low-frequency power/high-frequency power; SD: standard deviation

		LF power	HF power	LF/HF
Delirium group (n=14)	Average (SD) (ms^2^)	144 (201)	53.4 (74.7)	4.5 (2.8)
Non-delirium group (n=22)	Average (SD) (ms^2^)	326 (729)	257 (516)	3.0 (2.9)
Mann–Whitney U test	P-value		<0.05	
Normal value	Average (SD)	1170 (416)	975 (203)	1.5-2.0

## Discussion

We found a correlation between preoperative HRV values and postoperative delirium in patients aged 65 years or older undergoing scheduled open-heart surgery. HF is considered as the activity of the parasympathetic nerves. The onset of postoperative delirium may be associated with the preoperative functional decline of autonomic nerves, especially parasympathetic nerves.

We previously reported that preoperative HF is correlated with postoperative delirium in patients undergoing radical surgery for esophageal cancer [8]. The surgery for esophageal cancer is long and highly invasive, and the incidence of postoperative delirium was 23% [8]. A short-term preoperative HRV consistently predicts postoperative delirium across facilities and surgical sites.

Although whether low HF is associated with postoperative delirium is still a matter of debate, there is also a previous report that correlated autonomic nervous function with delirium [13], predicting susceptibility to postoperative delirium based on the non-invasive preoperative ECG measurements before surgery. Some recent papers have shown that HRV impacts postoperative complications and prognosis [14,15]. A study on the prognosis of coronavirus disease 2019 (COVID-19) patients based on HRV [16] concluded that a decrease in the activity of the autonomic nervous system due to sympathetic nerve depletion (e.g., the low standard deviation of RR intervals) and dominance of the parasympathetic nervous system (e.g., low normalized HF component) were associated with poor prognosis, increased mortality, and elevated interleukin 6 levels [16]. This way, the ability to predict prognosis based on information obtained from a nearly non-invasive ECG measurement is highly beneficial.

Open heart surgery has always been associated with a high incidence of delirium, and medical personnel works day and night to reduce the incidence of delirium. Initiatives are being taken to improve the surgical environment. However, the incidence of postoperative delirium is still high due to the highly invasive nature of the surgery, the need to stay in the ICU postoperatively [5,7], and the stress imposed on the patient by intubation and invasive devices. After the transfer to ICU, fentanyl and dexmedetomidine are used when adequate sedation is in effect, but sedation is discontinued when evaluation for delirium is initiated, and hence the impact of sedative medicine is not considered. Acetaminophen-based analgesic bases have been used in all cases. In this study, the incidence of postoperative delirium following planned open-heart surgery in patients aged 65 years or older was 39%, consistent with previous reports [7].

Length of ICU stay and cardiopulmonary time were examined in this study, and both of these were longer in the delirium group. Since it is clear that delirium prolongs the length of ICU stay [17], it is possible that delirium increased the length of ICU stay in our patients, or that there were many factors that caused delirium, such as postoperative infection. The fact that the duration of cardiopulmonary bypass surgery tended to be longer in the delirium group may also be a factor that triggered postoperative delirium. Regarding blood pressure during cardiopulmonary bypass, the target value was set the same in all cases, and there was no difference. Blood pressure monitoring during cardiopulmonary bypass was practiced based on past experience, and there was no difference between the delirium and the non-delirium groups. Recently, there has been a report that postoperative delirium can be reduced by adjusting blood pressure during cardiopulmonary bypass so that it does not fall below the lower limit of autoregulation capacity through real-time personalized brain monitoring [18]. We want to make it a matter for future consideration.

Postoperative delirium is said to develop due to a complex combination of factors such as depth of anesthesia, dehydration, pain, and anxiety [19]. Those elements have been excluded as much as possible. The patients were BIS-guided at a constant depth of anesthesia [20], and after undergoing similar anesthesia, were transported to the ICU under sedation. After the surgery, there was no bleeding, and the general condition improved; the sedation was cut off and the patients woke up. The pain was also quantified on the visual analog scale and differences were minimized. However, the possibility that these factors are involved in the onset of delirium due to individual differences cannot be denied.

In this study, delirium was diagnosed by CAM-ICU and not based on questionnaires from a psychiatrist. In the previous paper, we investigated the relationship between postoperative delirium and HRV in esophageal cancer surgery in the ICU, and delirium was diagnosed by psychiatrists [8]. Although this method is more popular because the ICU nurses evaluate during their working hours, unlike a psychiatrist's diagnosis, there is a high likelihood of overlooking low activity and reduced judgment accuracy [21]. Since there are only a limited number of facilities where psychiatrists are constantly available for evaluation in intensive care settings, we used the more common CAM-ICU method. The correlation found between HF and delirium, even in the CAM-ICU-based judgment, is very significant, and evaluation of the onset of delirium, which could be considered equivalent, was possible by CAM-ICU.

Since age is the most important factor in delirium onset, we limited the analysis to patients aged 65 years and older. Therefore, this study involved age groups prone to delirium in the clinical setting. Preoperative HRV is only a reflection of the preoperative condition. HRV cannot predict any significant event that may occur during the surgery. However, it can be considered an additional risk factor in terms of events that predict the risk of delirium, such as age, emergency surgery, surgical invasion, and electrolyte imbalance.

This study has a few limitations, primarily the fact that this was a single-center study; also, the sample size was relatively small and the observation period was short.

## Conclusions

Postoperative delirium is one of the most common and serious postoperative complications. Predicting postoperative delirium can provide better treatment for surgical patients. Preoperative HRV measurement is minimally invasive and might be helpful in predicting postoperative delirium after elective open-heart surgery in patients aged 65 years or older.
